# Nonlinear changes in the activity of the oxygen-dependent demethylase system in *Rhodococcus erythropolis *cells in the presence of low and very low doses of formaldehyde

**DOI:** 10.1186/1753-4631-5-9

**Published:** 2011-11-21

**Authors:** Elżbieta Malarczyk, Marzanna Pazdzioch-Czochra, Marcin Grąz, Janina Kochmańska-Rdest, Anna Jarosz-Wilkołazka

**Affiliations:** 1 Biochemistry Department, UMCS, Lublin, Poland

**Keywords:** formaldehyde, O-demethylases, homeopathy, *Rhodococcus erythropolis*, low doses

## Abstract

The effect of exogenous, highly diluted formaldehyde on the rate of demethylation/re-methylation of veratric acid by the bacteria *Rhodococcus erythropolis *was studied using electrophoretic and microscopic techniques. The activity of 4-O-demethylase, responsible for accumulation of vanillic acid, and the levels of veratric and vanillic acids were determined using capillary electrophoresis. Formaldehyde was serially diluted at 1:100 ratios, and the total number of iterations was 20. After incubation of the successive dilutions of formaldehyde with the bacteria, demethylase activity oscillated in a sinusoidal manner. It was established using capillary electrophoresis that methylation of vanillic acid to veratric acid occurred at a double rate, as shown by the doubled fluctuation in the concentration of veratrate. There were also changes in the NADH oxidase activity, which is associated with methylation processes. Microscopic observations revealed the presence of numerous enlarged vacuoles in bacterial cells during the accumulation of large amounts of vanillic acid, and their disappearance together with a decrease in 4-O-demethylase activity. The presented results give evidence for the ability of living cells to detect the presence of submolecular concentrations of biological effectors in their environment and provide a basis for a scientific explanation of the law of hormesis and the therapeutic effect of homeopathic dilutions.

## Introduction

Demethylation processes are widespread in all living organisms. They regulate replication and translation processes *via *methylation and demethylation of histones. In bacteria and fungi, they take an active part in transformations of phenolics, which form the basis of synthesis and biodegradation of lignin in plant tissues and humic acids in soil. Transformations of phenolics are strongly associated with secondary metabolism.

**Figure 1:**
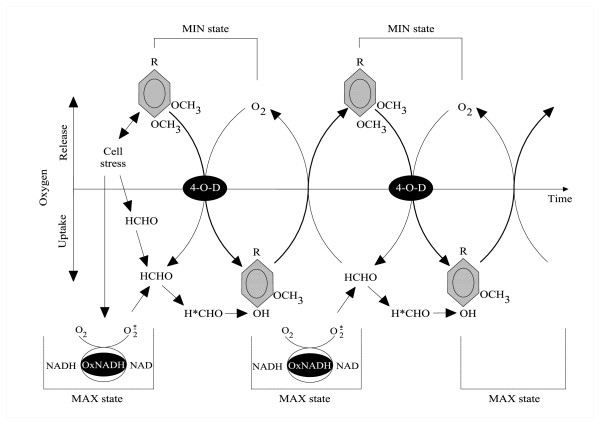
**The hypothetical mechanism of veratrate demethylation to vanillate by *Rhodococcus erythropolis *cells, dependent on activated state of formaldehyde (Malarczyk and Pazdzioch-Czochra, 2000) **[17]. Reproduced with permission of the Biochemical Society. Modified. Abbreviations: HCHO and H*CHO-formaldehyde in normal and activated state; 4-O-D-4-O-Demethylase system, Ox.NADH-NADH oxidase system, MAX state-maximal oxygen uptake, MIN state-maximal oxygen release. More details in the text of the original paper.

Methylathion is based on a reversible substitution of hydrogen with a -CH_3 _group at an electrophilic atom of nitrogen, oxygen, or sulfur. Demethylation refers primarily to the removal of a -CH_3 _group from a methoxy group (O-CH_3_), an N-methyl group (N-CH_3_) or an S-methyl group (-S-CH_3_), which leads to the liberation of formaldehyde (FA) [1]. During distribution of methyl groups in numerous cellular processes in animals, plants, and bacteria, an important role is played by enzymes, which facilitate the transfer of the one-carbon (-CH_3_) radical. The main substrates for these processes are the amino acids arginine and lysine [2]. Proteins rich in these amino acids, such as histones, owing to the proper distribution of methyl groups in them, determine the direction of many vital reactions, such as DNA activation [3,4]. Methylation of other proteins is responsible for chemotaxis in bacteria [5]. Also very important are transformations of natural catecholamines in higher animals, in which methylation of phenolic substances serves life-sustaining bioinformation purposes. It is phenolics and their methylated counterparts, methoxyphenols -- which under aerobic conditions undergo enzymatic catalysis with the participation of O-demethylases -- that constitute an important group of substrates for methylation and demethylation processes in bacterial and fungal cells [6-8]. Demethylase systems cooperate with a whole range of co-factors, including the FA molecule, which initiates the transformations referred to as the formaldehyde cycle [9]. Due to its high reactivity, this one-carbon molecule appears as a product of demethylation and, simultaneously, as a substrate for methylation. In lignin-degrading and humus-forming bacteria, among them *Rh. erythropolis *[10], methylation/demethylation processes are used to obtain recalcitrant carbon compounds from wood and lignin and thus contributing to detoxification of the environment [11,12]. Very similar processes of degradation of veratrate, vanillate, and isovanillate with the cooperation of 3O- and 4O-oxygenases and NAD(P)H and HCHO have been described by Providenti et al. [13,14] for the bacteria *Comamonas testosteroni BR 6020 *(formerly *Pseudomonas*). In aerobic conditions, these processes often become cyclic, and FA and its active states seem to play an important regulatory role in them [15]. In 1998, in a study of transformations of methoxyphenols in fungi and ligninolytic bacteria [16], we described the cyclic process of demethylation/methylation of veratric acid and vanillic acids in *Rh. erythropolis *[17], coupled with the activity of membrane bound NADH oxidase [18] and periodic reconstruction of the pool of veratric acid as a result of alternate activation of methylase and demethylase activities in these bacteria combined with changes in the levels of oxygen uptake. Figure 1 shows a hypothetical mechanism for these processes.

As the diagram shows, a single addition of potassium veratrate to the bacterial cell culture of *Rh. erythropolis *was enough to initiate those cyclic transformations. We concluded that the presence of the hard-to-assimilate veratric acid triggered in the investigated cells processes which were almost analogous to respiratory burst (MAX state in the diagram). During the violent oxygen uptake, NADH oxidase-mediated release of reactive oxygen species into the medium of *Rh. erythropolis *cells was observed. The ROS activated FA produced as a result of veratrate demethylation (Figure 1). Owing to this activation, FA became particularly reactive with vanillic acid and was able to convert it again into veratric acid *via *the path of non-enzymatic methylation. Thus, while demethylation is connected with the presence of an active form of the enzyme 4-O-demethylase, methylation seems to occur spontaneously using free-radical energy, which activates FA as a donor of a methyl group [19].

The periodic and spontaneous character of the observed changes in the activity of veratrate O-demethylases, measured as the amount of vanillic acid in the reaction medium, was clearly stimulated by the presence of FA and was associated with the NADH oxidase activity. The above relationships testify to the existence in the examined bacterial cells of autoregulatory mechanisms, the molecular aspects of which remain to be explained. Those findings became an incentive for further observations of the interrelationship between the two enzymes and the level of FA, this time with the focus on the exogenous effect of this compound. The experiments presented below were carried out on the basis of previous work on fungal oxidases [20], fungal laccase [21] and plant peroxidase [22,23], and the changes in their activities in the presence of low doses of phenolic co-factors. The present study provided new data relating to the influence of different FA dilutions on changes in the activity of veratrate O-demethylase and the cooperating NADH oxidase in the cells of *Rh. erythropolis*.

## Materials and methods

### Biological material

Cells of *Rhodococcus erythropolis *(previously Nocardia sp DSM 1069) were cultivated on a liquid medium according to [17]. Cultures were grown in 250 ml conical flasks filled with 100 ml medium on a rotary shaker at 30°C. The cells were collected in the mid-logarithmic phase of growth by centrifugation at 10 000 × g, and a suspension of optical density A660 = 1 was prepared with 0.06 M phosphate buffer, pH 7.4.

### Formaldehyde dilutions

A 1 M solution of FA in phosphate buffer, pH 7.4, was diluted at a 1:100 ratio in distilled water and in 75% ethanol by transferring 0.1 ml of each successive FA dilution into 9.9 ml of diluents and dynamically shaking [22,23]. After 20 transfers, two series of dilutions of FA solutions, named from n = 1 to n = 20, were obtained: one in distilled water and the other in 75% ethanol. As a control, two independent series of pure water and 75% ethanol were prepared by shaking, in an analogous manner to that described for the dilutions of formaldehyde.

After n transfers (dilution steps) the FA concentration is equal

(1) $${\text{c}}_{\text{n}}={\text{c}}_{0}∕{\text{r}}^{\text{n}}$$

where

c_0 _denotes initial concentration, and r denotes dilution ratio.

In our case c_0 _= 1 M and r = 100 we have

(1a) $${\text{c}}_{\text{n}}=10^{-2\text{n}}$$

So, n is a unit of the logarithmic concentration scale

(2) $$\text{n}=-\left[\log\left({\text{c}}_{\text{n}}∕{\text{c}}_{0}\right)∕\log\left(\text{r}\right)\right]$$

Here we have log(r) = log(100) = 2 and

(2a) $$\text{n}=-\log\left({\text{c}}_{\text{n}}∕1\right)∕2=-1∕2\log\left({\text{c}}_{\text{n}}\right)$$

with concentration c_n _in mol/liter.

In our case only integer values of n make sense. So we have to stress that in all our Figures the wave-like lines are drawn only for convenience to indicate the points belonging to a given series.

### Incubation of FA dilutions with Rh. erythropolis cells

A cell suspension with an optical density of 1 at 660 nm was supplemented with veratric acid (200 μl 2% acid per 10 ml suspension) and divided into twenty one 10 ml aliquots. Each aliquot was then supplemented with 200 μl of a selected dilution of FA in ethanol, water, or an appropriate control. The suspensions were left to stand at room temperature for a variable period of time, dependent on the type of experiment. After incubation, the samples were centrifuged, and the cell pellet was separated from the supernatant. The cell pellet was used to determine NADH oxidase activity, and the supernatant was used to determine the level of phenolic acids.

### Determination of demethylase activity in a colorimetric test

In the supernatant, after centrifugation of cells, two main products of veratrate demethylation, vanillic and isovanillic acids, were determined in a colorimetric test with DASA (diazosulphamide) [10]. 0.2 ml of each sample was mixed with 0.2 ml of 2% sulfanilamide solution in 10% hydrochloric acid followed by 0.2 ml of 5% NaNO_2_. After 1 min, the reaction mixture was neutralized with 1 ml of 20% Na_2_CO_3_, and absorbance at 500 nm (for vanillic acids) was measured. The amount of vanillic acids was calculated based on a calibration curve (y = 6.85 x - 0.0218, R2 = 0.999). All the measurements were done in triplicate. Data were obtained for the same set of samples.

### Capillary electrophoretic determination of phenolic substances

Because the colorimetric test provided no information on the qualitative and quantitative composition of the mixture of phenolic substances, the supernatant, after separation of *Rh. erythropolis *cells, was used to establish the amount and type of the phenolic products of demethylation using micellar electrokinetic chromatography (MEKC). Analyses of the supernatants obtained after incubation of the water and ethanol dilutions of FA with *Rh. erythropolis *cells (see Biological Material) were performed using Thermo Capillary Electrophoresis, Crystal 100, (Thermo Separation Products, San Jose, USA) equipped with a UV-Vis diode array detector. Separations were carried out using a 50 μm ID fused silica capillary with a total length of 70 cm (44 cm to the detection window). The applied voltage was 29 kV, and the capillary temperature was maintained at 30°C. Samples were injected hydrodynamically for 1 second, and detection was performed at 210 nm. Separation was done in a buffer prepared by dissolving boric acid (100 mM) and sodium dodecyl sulfate (SDS, 100 mM) in MilliQ water. The buffer, pH 9.3, was adjusted using NaOH. Peak identification was done by spiking with commercially available veratric, vanillic, isovanillic, and protocatechuic acids and catechol, as potential products of demethylation. The capillary was conditioned using 1 M NaOH by 10 min. and 5 min. washing with 0.1 NaOH before filling with buffer at the start of each day of analysis. Before each analysis, the capillary was conditioned with 1 M NaOH, 0.1 M NaOH, MilliQ water, and filling buffer, 2 min. each.

### Determination of NADH oxidase activity

The NADH oxidase activity was determined for the cell membrane fraction, following homogenization of cell pellet in a chilled mortar after centrifugation of 20 ml of cell suspension. The membrane pellet was resuspended in 0.4 M acetate buffer, pH 5.0. The activity was determined by measuring oxygen uptake with an oxygen electrode, according to Vianello and Macri [24], in the presence of NADH. A pellet of cells which had no contact with veratrate was used as a control. 400 mM of acetate buffer, pH 5.5, was mixed with 1.26 M saccharose and 0.5 ml of sample, and 4 mM NADH was added at the start. The NADH oxidase activity was expressed as oxygen uptake (μM/ml).

### Electron microscopy

Electron microscopy was carried out after embedding the cells in a mixture of 2% paraformaldehyde and 2.5% glutaraldehyde in cacodylic buffer, pH 7.4. For a better contrast, 1% OsO_4 _in cacodylic buffer and 0.5% uranyl acetate in water were used. The cells were dehydrated in ethanol (30% to 100% ethanol solutions) at room temperature and exposed to a propylene oxide concentration series, changing gradually from alcohol into pure oxide.

Spurr Low Viscosity resin was used to prepare ultrathin sections on an ultramicrotome (Reichert Ultracut S). Observations were made using a Tesla BS-500 microscope.

## Results

### The course of demethylation of veratric acid

**Figure 2:**
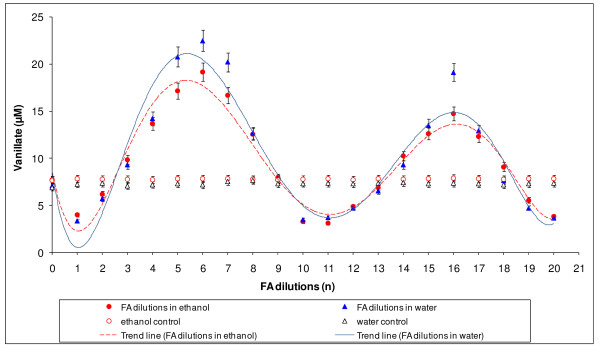
**The demethylation of veratric acid by *Rhodococcus erythropolis *cells after 6-hour incubation with successive FA dilutions in water or in 75% ethanol, together with control values for water and 75% ethanol**.

Demethylation of veratric acid by *Rhodococcus erythropolis *turned out to be a diverse process, both in terms of the formaldehyde dilutions used and the time of incubation of cells with the substrate. The results provided clear evidence that the level of demethylation varied quantitatively depending on the dilution of formaldehyde. The curve obtained for water dilutions of FA showed slightly higher demethylation values than an analogous curve for ethanol solutions. Values obtained for both controls indicated no changes in demethylase activity (Figure 2). For both diluents, maximum demethylation was observed at formaldehyde dilutions n = 6 and n = 16, and minima corresponded to dilutions n = 1, n = 10-11, and n = 20. In 10-hour incubation experiments, only the ethanol series of FA dilutions was used so that sterile conditions could be maintained.

**Figure 3:**
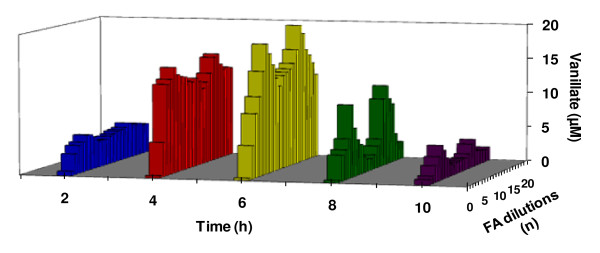
**Demethylation of veratric acid by *Rhodococcus erythropolis *cells in the presence of low doses of HCHO as a function of time**. Planar version (2D).

**Figure 4:**
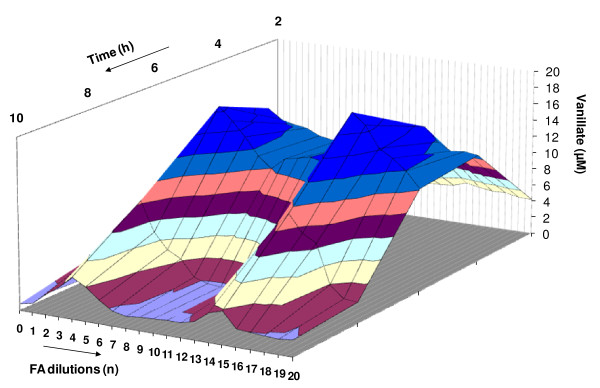
**Demethylation of veratric acid by *Rhodococcus erythropolis *cells in the presence of low doses of HCHO as a function in time**. Spatial version (3D).

In a following experiment, the optimal time of incubation was determined for maximally and minimally active dilutions of the ethanol series. For this purpose, samples of incubating suspensions were taken every two hours, with measurements being done for 10 hours. The activity over time are shown in a 2D version (Figure 3) and a 3D version (Figure 4).

The results showed that the most effective time for demethylation of veratric acid by *Rhodococcus erythropolis *was 6 hours, for both activating dilutions (n = 5 and 6) and inhibiting ones (n = 15 and 16) (Figure 3 and 4).

### Qualitative and quantitative determination of demethylation products

**Figure 5:**
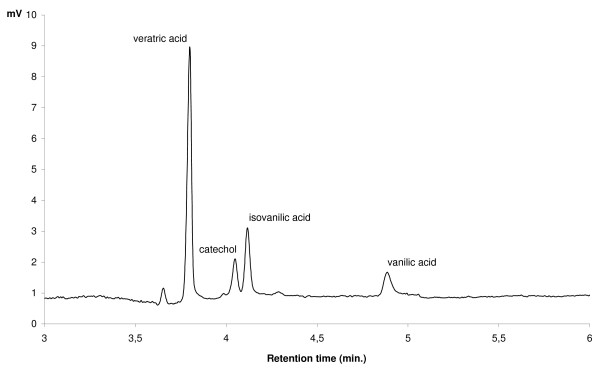
**Chromatogram (MEKC) of veratric, vanillic, isovanillic acid and catechol standards**.

**Figure 6:**
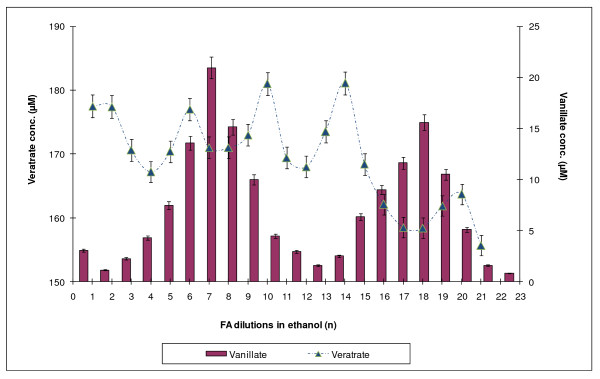
**Changes in the level of veratric acid during incubation of formaldehyde with *Rh. erythropolis *cells in a series of HCHO dilutions in ethanol and the corresponding levels of vanillic acid identified using MEKC**.

**Figure 7:**
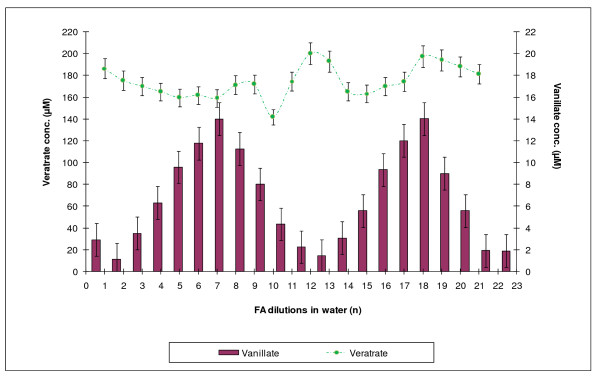
**Changes in the level of veratric acid during incubation of formaldehyde with *Rh. erythropolis *cells in a series of HCHO dilutions in water and the corresponding levels of vanillic acid identified using MEKC**.

DASA is used to determine the amounts of those products of veratrate demethylation which have free OH groups, but does not allow one to qualitatively discriminate among them or to measure the concentration of veratric acid, which does not contain phenolic groups and, therefore, cannot be registered by the diazosulfanilamide method. To obtain those data, capillary electrophoretic determination of phenolic substances was performed. The use of this method made it possible both to establish the level of veratric acid and to discriminate between the two vanillic acids (Figure 5). Measurements were done for both FA dilution series after 6 hours of incubation (Figure 6 and 7).

The MEKC measurement revealed the presence of only one product in the reaction environment -- vanillic acid. Neither isovanillic acid nor catechol, which theoretically could accompany demethylation of veratric acid, was found. Changes in the concentration of vanillic acid and corresponding concentrations of veratric acid are shown in Figures 6 and 7.

The nearly exclusive presence of vanillic acid in the incubation medium pointed to the type of demethylase which was activated as a result of incubation of *Rhodococcus *cells with selected formaldehyde dilutions. The enzyme was 4-O-demethylase, which converted veratric acid into vanillic acid. In experiments with dilutions of the ethanol series, there appeared a clear quantitative relationship between the periodic reconstruction of the pool of veratric acid and the amount of vanillic acid in the incubation medium. The double number of veratric acid maxima pointed to the double frequency of methylation of vanillic acid, compared with demethylation of veratric acid (Figure 6). Incubation of cells with FA dilutions in the water series caused more chaotic changes in the level and the renewal of veratric acid (Figure 7). Nonetheless, the character of the changes remained the same.

### Determination of NADH oxidase activity in Rh. erythropolis cells

**Figure 8:**
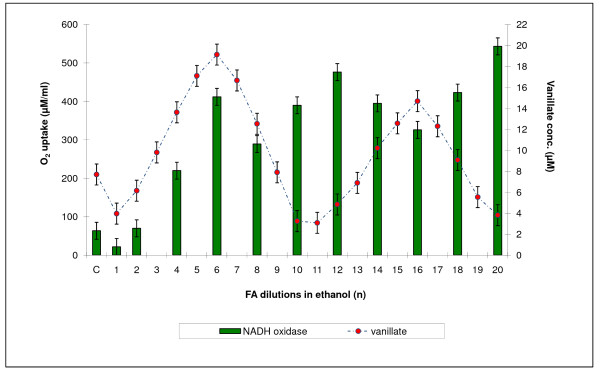
**A comparison between demethylation of *Rh. erythropolis *cells and their oxygenase activity against NADH**. Ethanol series.

**Figure 9:**
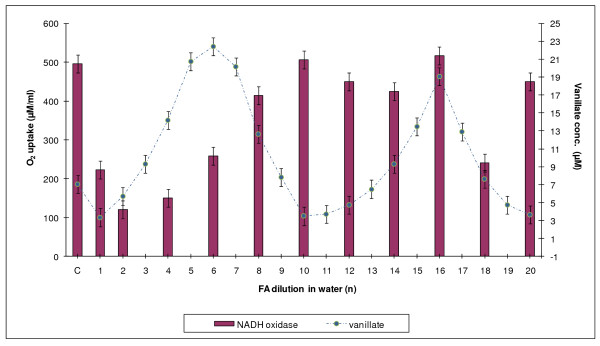
**A comparison between demethylation of *Rh. erythropolis *cells and their oxygenase activity against NADH**. Water series.

Changes in NADH oxidase activity were observed during incubation of *Rh. erythropolis *cells with both the water and the ethanol HCHO dilution series. For easier comparison of the distribution of the maxima and minima of demethylase and oxidase activities, the level of NADH oxidase activity was plotted in Figure 8 and 9 against DASA values from Figure 2 corresponding to demethylase activity. It was found that oxidase oscillations were less regular than demethylation processes. It was also observed that not all maxima were positioned opposite one another. A particular lack of regularity was observed in the action of the dilutions of the water series.

### Analysis of morphological changes in Rh. erythropolis cells under the influence of high HCHO dilutions

**Figure 10:**
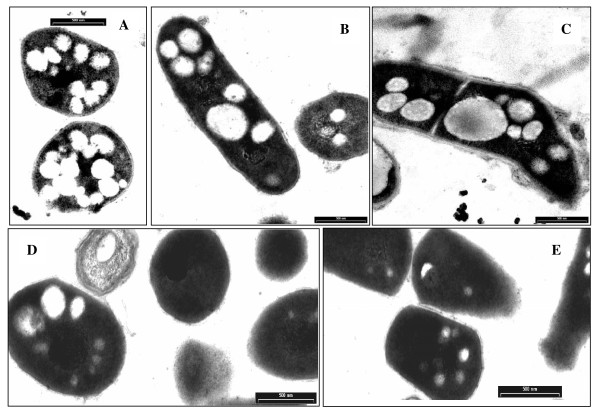
**Differences in morphology of *Rh. erythropolis *cells under the action of very low doses of formaldehyde in 75% ethanol**. A-control with ethanol; B and C-maximum of demethylation, n = 5 and 15; D and E-minimum of demethylation, n = 10 and 20.

A comparison was made among microscopic images of the morphology of *Rh. erythropolis *cells incubated with selected FA dilutions (Figure 10). The images provided further information on the effect of HCHO dilutions on the morphology of the bacterial cells. It was observed that the dilutions with maximum potency increased the volume and size of intracellular vacuoles, in contrast to the least potent dilutions, at which the vacuoles tended to disappear. The control cell material, which had no contact with veratric acid, showed the presence of a large number of small vacuoles, which markedly differed from the more numerous and considerably larger vacuoles found in the cells incubated with the FA dilutions showing maximum 4-O-demethylase activity. This state differed clearly from the image of cells with a low enzymatic activity, where vacuoles were restructured and tended to disappear.

## Discussion

To investigate the regulatory properties of formaldehyde in demethylation processes, a model system of demethylation of veratric acid by *Rh. erythropolis *cells, described in our earlier papers [17,19], was used. This biological system is sensitive both to an excess and a lack of FA as well as to normal and excited states of this molecule. The system pointed to 4-O-demethylase as the enzyme responsible for the accumulation of vanillic acid in the incubation medium. The experiments described above showed that the presence of diluted FA in the reaction medium significantly affected the rate of demethylation. The capillary electrophoretic experiments (MEKC) conducted in this study demonstrated that the accumulation of vanillic acid was changed. On the basis of these results, it was possible to choose those FA dilutions which clearly activated or inhibited the activity of 4-O-demethylase and were, accordingly, recorded as maximum or minimum concentrations of vanillic acid in the bacterial culture.

MEKC also enabled a comparison between the concentrations of vanillic and veratric acids. The fluctuations in the concentration of veratric acid showed which FA dilutions caused its renewal. As discussed in our previous paper, the pool of veratric acid is reconstructed spontaneously as a result of non-enzymatic methylation of vanillic acid with the participation of excited FA [17]. Excitation of FA occurs with the participation of reactive oxygen species as a result of veratric acid coming in contact with the bacterial system of NADH oxidase, which generates free radicals (see also [25-27]. In the present study, similar effects of activation of 4-O-demethylase and distinct changes in NADH oxidase were obtained by using appropriate FA dilutions prepared by successive iterations in 75% ethanol connected with shaking. It is highly probable that shaking (i.e., enhancing the potential energy of the system) has an effect on the state of excitation of FA, since both 4-O-demethylase and NADH oxidase were activated during incubation with *Rh *cells (Figure 1).

The occurrence of the successive maxima of activity, which was a consequence of increasing dilutions,, indicated that the investigated effect was repeatable. Similar relationships had been described earlier for fungal peroxidase and laccase [20], and HR-peroxidase [22,23]. In the present study, the plot of activation of 4-O-demethylase by selected FA dilutions was supplemented with the parameter of time. In two-dimensional (2D) space (Figure 3) was now transformed into a system with a distinctly varied surface visualized in 3D (Figure 4). Observations over time pointed to dynamic aspects of the described changes. They showed that maximum activation of 4-O-demethylase as a result of incubation of selected FA dilutions with *Rh*. cells was achieved after 6 hours. Due to the dynamic character of those transformations, the course of demethylation over time resembled the effects described by Calabrese and Baldwin [28,29] as hormesis.

Especially one of the latest works of Calabrese et al. [30] and Szende et al. [31] are worth discussing in the context of the present studies. These two works demonstrate the effect of applying appropriately chosen low doses of resveratrol, now a commercially available diet supplement. Resveratrol is commonly found in nature, e.g., large amounts of it are present in red grapes. Resveratrol is a natural stilbene in a trans configuration, which shows strong detoxifying properties. Therefore, it is very probable that these detoxifying effects are the result of intensive elimination of FA [32] even when resveratrol is present in very low doses [31]. The results obtained in the present study prove that also the enzymes connected with methylation/demethylation metabolism, 4-O-demethylase and NADH oxidase, are sensitive to low and very low doses of FA.

Just as in our previous papers [22,23], it was demonstrated in this study that the effect of diluted effectors has a fractal character and their periodic action is independent of the Avogadro constant. If we had not exceeded this value, we would not have been able to demonstrate the presence of further maxima of occurrence of vanillic acid, which required further dilution beyond 10^-24 ^(Figure 2). Using dilutions ranging from C1 (100^-1^) to C20 (100^-20^), we were able to show two maxima of accumulation of vanillic acid at a 10-iteration interval (20 decimal places). After those 10 steps, the level of activity returned to the previous state. This suggests the existence of a specific cycle for those processes requiring 10 iterations. The fluctuations in demethylation activity bear a considerable resemblance to the frequency of the transformations observed previously for the action of phenol on the activity of plant peroxidase HRP [22,23] and fungal laccase [20]. It is also important to note that methylation processes reproducing veratric acid occur more frequently-every five iterations (Figure 6).

Active FA dilutions also triggered a response in live *Rh. erythropolis *cells. When coming in contact with very low doses of FA, the cells reacted with clear changes in their morphology, both with respect to the quantity and quality of vacuoles. The number and size of the vacuoles was visibly higher in cells incubated with those FA dilutions which stimulated a high 4-O-demethylase activity. This was connected with an increase in the concentration of vanillic acid in the incubation medium. At the same time, minimum states of demethylase activity correlated in the cells with the disappearance of vanillate and simultaneous renewal of veratric acid and were characterized by a drop in the number of vacuoles. Almost the same relationships had been described in an earlier study [17], in which the number of vacuoles was shown to increase in cells which released large amounts of oxygen (oxidative burst). This was also connected with a decrease in the amount of vanillic acid. The present data confirmed the previous finding that the demethylation processes occurring during the use of FA dilutions strictly correlated with changes in cell morphology.

In both this and our previous studies, formaldehyde seemed to be a significant regulator of demethylation. However, while in the earlier papers the regulatory effect of FA had been induced endogenously, here FA was used as an exogenous factor of variable concentration. Whereas previously demethylation had been initiated by a single addition of veratric acid to a non-growing bacterial culture, in the present study, the same process depended on adding an appropriate FA dilution. Inasmuch as the morphological changes in the cells after a single addition of veratric acid to a bacterial culture are a logical consequence of the gradual decrease in the concentration of substrates in the incubation medium during the reaction, the observation that this process can be carried on by using appropriate FA dilutions as co-substrates seems to be a prelude to acknowledging that living cells can detect the presence of submolecular concentrations of biologically active substances. FA is easily excited as a result of both coming in contact with reactive oxygen species [33,34] and energetic changes in the system [35]. Among reactive oxygen species, H_2_O_3 _seems to be a particularly interesting molecule [36,37]. Its presence could be reflected by changes in a number and size of vacuoles, increased after the treatment of *Rhodococcus *cells with the selected formaldehyde dilution.

The novelty of this work is that it provides further experimental evidence for the possibility of inducing a biological effect by exposing living cells to the action of highly diluted chemical substances. The mechanism of this phenomenon has not yet been elucidated but according to the papers of Montagnier et al. [38], serial dilution of substances in water correlated with agitation can generate electromagnetic signals in these solutions. Montagnier's experiments with high aqueous dilutions of some bacteria and HIV revealed that DNA and some proteins induced the emission of electromagnetic waves [38]. This was connected with the induction of the emission of electromagnetic waves [38].

We also provided some experimental evidence for this phenomenon. We showed that by carrying out experiments with a series of successive effector dilutions, one can effectively choose those dilutions which have a distinctly strong biological action. This possibility of using a non-linear phenomenon to predict the behavior of model biological systems leads to rational design of new research systems and their verification on the basis of the ones that already exist. Such a double verification of the results is a domain of synthetic biology, which places a large emphasis on rational design of new systems and intensive use of model techniques for predicting the behavior of systems and optimization of their operation.

The changes in time of the solution's n = 0 activity are not clear yet and an autooscillatory reaction can be taken into the consideration. In the future we are going to test n = 0 solution to check a possibility of autooscillatory nature of such a reaction.

## Competing interests

The authors declare that they have no competing interests.

## Authors' contributions

EM projected and coordinated the experiments. MPC conducted the NADH oxidase activity assay. MG performed MEKC analysis. JKR prepared the biological material and conducted the demethylase activity assay. AJW participated in the sequence alignment. All authors read and approved the final manuscript.
